# Bioactive sphingolipid profile in a xenograft mouse model of head and neck squamous cell carcinoma

**DOI:** 10.1371/journal.pone.0215770

**Published:** 2019-04-19

**Authors:** Aiping Bai, Xiang Liu, Jacek Bielawski, Yusuf A. Hannun

**Affiliations:** 1 Department of Biochemistry & Molecular Biology, Medical University of South Carolina, Charleston, South Carolina, United States of America; 2 Lipidomics Shared Resources, Medical University of South Carolina, Charleston, South Carolina, United States of America; 3 Department of Microbiology & Immunology, Medical University of South Carolina, Charleston, South Carolina, United States of America; 4 Departments of Medicine and Biochemistry & the Stony Brook Cancer Center at Stony Brook University, Stony Brook, New York, United States of America; Universite Paris Diderot-Paris7 - Batiment des Grands Moulins, FRANCE

## Abstract

The purpose of this study was to determine the profile of bioactive sphingolipids in xenograft mouse tissues of head and neck squamous cell carcinoma. We utilized UHPLC-MS/MS to determine the profile of full set of ceramides, sphingosine, and sphingosine 1-phosphate in this xenograft mouse model. The tissues isolated and investigated were from brain, lung, heart, liver, spleen, kidney, bladder, tumors and blood. With the exception of equal volume of blood plasma (100ul), all tissues were studied with the same amount of protein (800ug). Results demonstrated that brain contained the highest level of ceramide and kidney had the highest level of sphingosine, whereas sphingosine 1-phosphate and dihydrosphingosine 1-phosphate were heavily presented in the blood. Brain also comprised the highest level of phospholipids. As for the species, several ceramides, usually present in very low amounts in cultured tumor cells, showed relatively high levels in certain tissues. This study highlights levels of bioactive sphingolipids profiles in xenograft mouse model of head and neck squamous cell carcinoma, and provides resources to investigate potential therapeutic targets and biomarkers that target bioactive sphingolipids metabolism pathways.

## Introduction

Bioactive sphingolipids (SL), which include ceramides (Cer), sphingoid bases, and their phosphates, make up the early products of the SL synthetic pathways. Cer, the central molecule, is associated with the action of several growth suppressor stimuli and inflammatory signals [1–3]. Cer can either be produced from complex SL or be synthesized (*de novo* pathway) from dihydroceramide (dhCer) under the catalysis of dhCer desaturase (DES1) [4]. Sphingoid bases are the fundamental building blocks of all SL. The main mammalian sphingoid bases are dihydrosphingosine (dhSph) and sphingosine (Sph). Sph has functional roles in regulating the actin cytoskeleton, endocytosis, cell cycle and apoptosis [5–6]. Cer can be hydrolyzed by ceramidase (CDase) to produce Sph. Sph is subsequently phosphorylated by Sph kinases (SKs) to generate Sph 1-phosphate (Sph 1-P), and Sph 1-P has a critical role in many physiological and pathophysiological processes, such as atherosclerosis, diabetes, and cancer et al [7–9].

Head and neck squamous cell carcinoma (HNSCC) is the most common head and neck cancer, and is widely known to be resistant to many kinds of treatments (chemotherapy, radiation, and surgery, et al) [10]. Previously, our group and others uncovered targeting Cer metabolism enzymes, such as DES1, ACDase, SK1, as wells as certain chain length of Cer could sensitize resistant cells to various therapies and improve HNSCC cell killing [11–15]. Therefore, HNSCC xenograft mouse model is a very efficient model to validate efficacy and side effects of such enzyme inhibitors. However, the profile of bioactive SL in xenograft mouse model has not been fully described yet. In this study, we utilized ultra-high performance liquid chromatography tandem mass spectrometry (UHPLC-MS/MS) to determine the profile of bioactive SL, and we provide the basal levels of Cn-Cer (ceramide species with n carbons in the fatty acyl chain), Sph, Sph 1-P, dhC16-Cer, dhSph and dhSph 1-P in xenograft mouse model of HNSCC. The tissues we isolated and investigated are from brain, lung, heart, liver, spleen, kidney, bladder, tumor and blood.

## Materials and methods

### Cell culture and reagents

The HNSCC cell line SCC-14a was maintained in DMEM medium with L-glutamine and 4.5g/l glucose (Media-tech, Herndon, VA). When prepared for *in vivo* studies, SCC-14a were seeded in a 150mm dish to reach around 70% confluence and harvested using cell stripper after washing with cold PBS twice, then centrifuged at 500g, and cells pellets were re-suspended in serum-free medium at concentration of 5x10^7^/ml.

### Animal studies

All procedures were performed according to guidelines of Medical University of South Carolina institutional biosafety committee (MUSC/IBC). Mice care/ welfare and experiments were carried out according to the approved protocol (AR3157, Bai A), Medical University of South Carolina Institutional Animal Care and Use Committee (IACUC). Briefly, nu/nu athymic nude mice were kept in a pathogen-free environment. Later, mice (at age of 8–9 weeks) were injected subcutaneously into the right flank with SCC-14a (5x10^6^/100ul). Mice were then monitored twice weekly for the tumor growth. When tumor appeared, tumor size was calculated using the formula [tumor volume (mm^3^) = π/6 *Length *Width *Depth]. Mice were enrolled in the experiment when established flank xenografts reached >100mm^3^, which is also the starting point for the drug candidates’ *in vivo* therapeutic validation. A total 6 mice was utilized in the studies.

### Samples preparation

Once qualified for the studies, mice were sacrificed, and tissues (lung, liver, brain, spleen, bladder, kidney, heart, and tumor) and blood (250ul) were isolated. Tissue (heart and bladder) was quickly dipped in cold PBS twice before being dried down. All tissues were quickly stored in liquid nitrogen for future protein isolation. Later, tissues were homogenized in lysis buffer containing protease-inhibitor cocktail before centrifugation at 12,000 g for 10 min (4°C) to get the supernatant for protein quantification. Then equal amount of protein (800ug) were provided for analysis of SL.

Blood samples were quickly put in sterilized 0.5ml Eppendorf tubes that were previously treated with 0.5M sterile EDTA (10ul), centrifuged at 2,000 g for 10min (4°C) to obtain plasma, and then 100ul equal volume of plasma provided for analysis of SLs.

### Lipid extract preparation and UHPLC-MS/MS analyses of SL

Lipids extracts were prepared, and advanced analyses of endogenous bioactive SL were performed as previously described [16]. SL levels were normalized to the total protein content (per mg) or equal volume (100ul).

### Statistical analysis

Where indicated, data are represented as mean ± SD. Statistical analysis was performed using two-sided *t* test, with *p*-value <0.05 considered statistically significant.

## Results and summary

### Comparison of SL in various tissues

**Fig 1:**
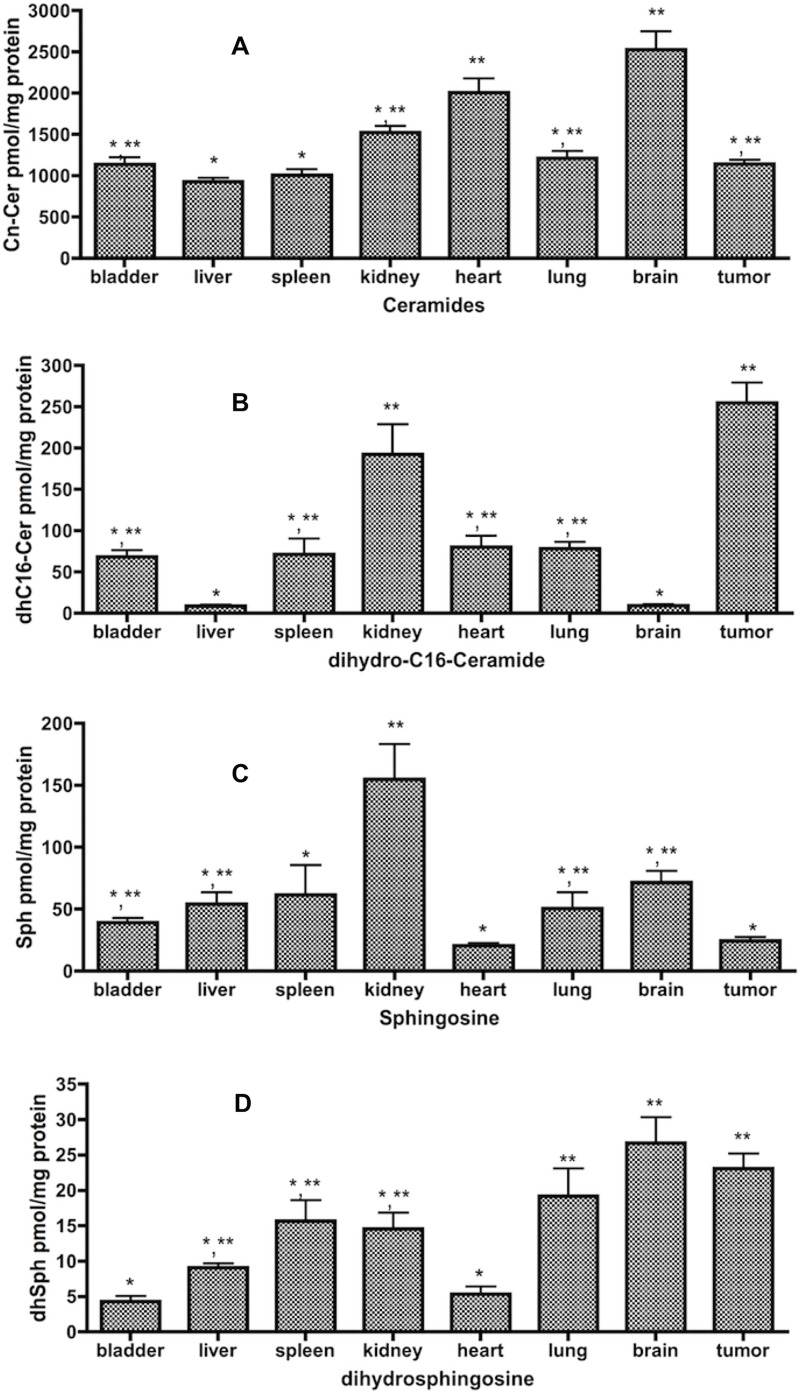
Levels of total Cer, Sph, dhC16-Cer, and dhSph present in various tissues. Tissues were homogenized in lysis buffer containing protease-inhibitor cocktail before centrifugation to get protein. Then equal amounts of protein (800ug) were provided for analysis of SL. Results are presented as pmol SL/mg protein with means ± st dev. of 6x replicates. **A**.Total Cer, * *p*<0.05 (*vs* brain), ** *p*<0.05 (*vs* liver); **B**.dhC16-Cer, * *p*<0.05 (*vs* tumor), ** *p*<0.05 (*vs* liver); **C**. Sph, * *p*<0.05 (*vs* kidney), ** *p*<0.05 (*vs* heart); **D**. dhSph, * *p*<0.05 (*vs* brain), ** *p*<0.05 (*vs* bladder).

Initially, we determined the levels of bioactive SL in tissues, blood, and tumor xenograft. The results (Fig 1 and Table 1**)** showed that among the eight tissues analyzed (lung, liver, brain, spleen, bladder, kidney, heart, and tumor), brain contained the highest level of Cer (2.53nmol), which was double the amount of Cer in most of the tissues that were analyzed (Fig 1A). Tumor xenograft contained the highest level of dhC16-Cer (254.8pmol) whereas the lowest levels of dhC16-Cer were found in liver (8.5pmol) and brain (9.2pmol), showing an almost 30 folds difference between the highest and lowest (Fig 1B). Kidney contained the highest level of Sph (154. 8pmol), while the lowest level of Sph was found in heart (20.5pmol) (Fig 1C). Brain also contained the highest level of dhSph (26.7pmol), and the lowest levels were in heart and bladder (4-5pmol, Fig 1D).

**Table 1:** Levels of total Cer, dhC16-Cer, Sph and dhSph in various tissues.

**Tissue (pmol** **/mg pro)**	**Total Cer**	**Sph**	**dhC16-Cer**	**dhSph**
Bladder	1139.7±206.0	39.1±9.5	68.5±19.0	4.3±1.9
Liver	926.0±108.7	54.2±22.5	8.5±4.6	9.1±1.4
Spleen	1006.6 ±178.8	37.8±5.7	71.5±46.3	15.7±7.2
Kidney	1522.1±202.5	**154.8±69.6**	192.0±64.4	14.6±5.5
Heart	2005.6±422.1	20.5±5.1	80.2±32.8	5.3±2.6
Lung	1214.0±210.1	50.4±32.3	78.4±19.6	19.2±9.5
Brain	**2529.9±534.3**	71.7±22.4	9.2±3.5	**26.7±8.8**
Tumor	1143.9±124.4	24.6±7.6	**254.8±60.4**	23.1±5.2

Results are presented as pmol SL/mg protein with means ± st dev. of 6x replicates.

**Fig 2:**
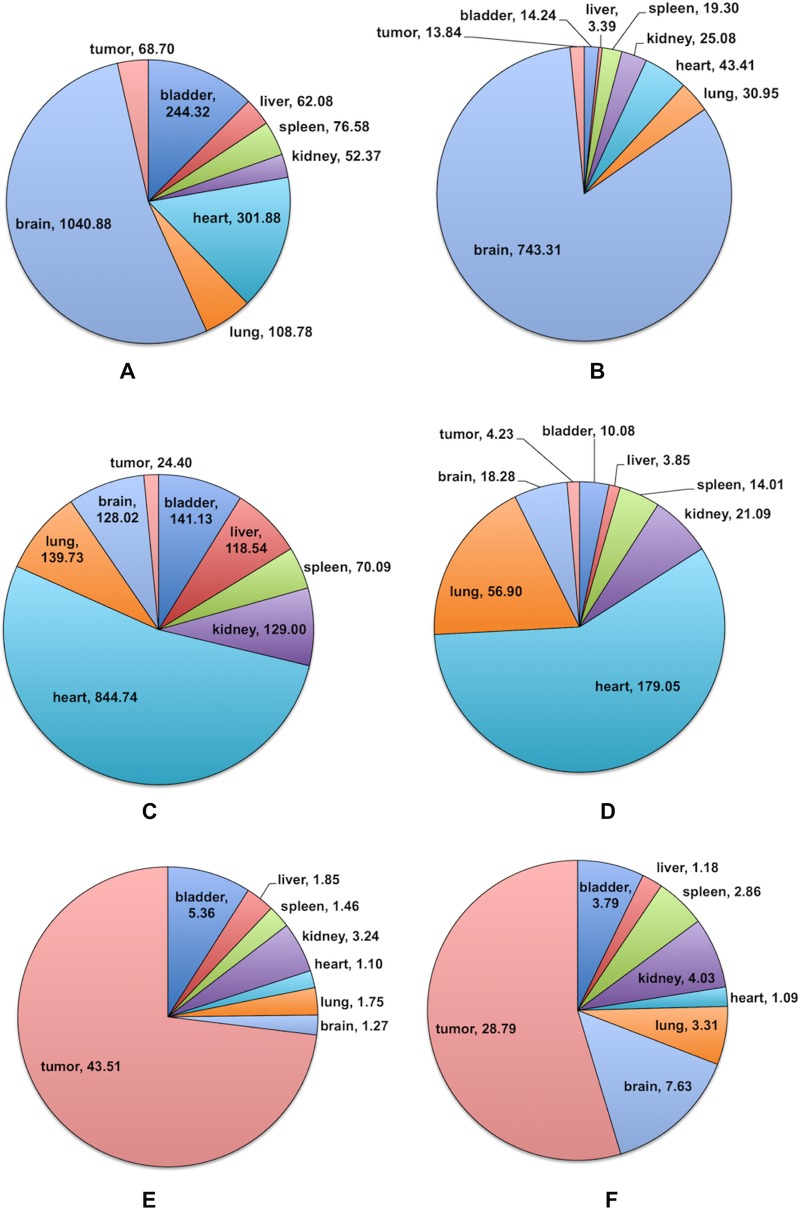
Predominant Cn-Cer species in eight tissues. Results are presented as pmol Cn-Cer/mg protein with means ± st dev. of 6x replicates. **A**. C18-Cer; **B**.C18:1-Cer; **C**. C20-Cer; **D**. C20:1-Cer; **E**. C26-Cer; **F**. C26:1-Cer.

As to the specific Cn-Cer species, interestingly, several Cn-Cer that usually present very low levels in cultured tumor cells showed relatively high levels in certain tissues. For example, C18 and C18:1-Cer were heavily present in the brain (Fig 2A and 2B and Table 2); C20 and C20:1-Cer were mainly in the heart (Fig 2C and 2D and Table 2). On the other hand, head and neck tumor contained the highest levels of C26 and C26:1-Cer, and the lowest level of these two species were in heart tissue (1pmol, Fig 2E and 2F and Table 2).

**Table 2:** Levels of C18, C18:1-Cer; C20, C20:1-Cer; C26, C26:1-Cer in various tissues.

Tissue (pmol /mg pro) *	**C18-Cer**	**C18:1-Cer**	**C20-Cer**	**C20:1-Cer**	**C26-Cer**	**C26:1-Cer**
Bladder	244.3±48.4	14.2±2.9	141.1±31.3	10.1±2.7	5.4±2.2	3.8±1.8
Liver	62.1±24.4	3.4±1.2	118.5±58.8	3.9±1.2	1.9±0.9	1.2±0.3
Spleen	76.6±30.0	19.3±3.1	70.1±37.5	14.0±1.8	1.5±0.8	2.9±1.2
Kidney	52.4±13.6	25.1±6.8	129.0±35.2	21.1±4.2	3.2±1.4	4.0±1.7
Heart	301.9±76.5	43.4±8.0	**844.7±252.3**	**179.0±30.0**	1.1±0.5	1.1±0.3
Lung	108.8±22.8	31.0±5.9	139.7±50.6	56.9±10.3	1.8±0.9	3.3±0.7
Brain	**1040.9±295.5**	**743.3±262.2**	128.0±26.1	18.3±4.1	1.3±0.6	7.6±2.5
Tumor	68.7±31.5	13.8±4.2	24.4±8.4	4.2±1.0	**43.5±10.1**	**28.8±8.9**

Results are presented as pmol Cn-Cer/mg protein with means ± st dev. of 6x replicates.

* All *p* values <0.001 (*vs* highlighted species).

### Cn-Cer profile in various tissues

Next, we performed a comparative analysis of Cn-Cer in the tissues studied.

#### Brain

**Fig 3:**
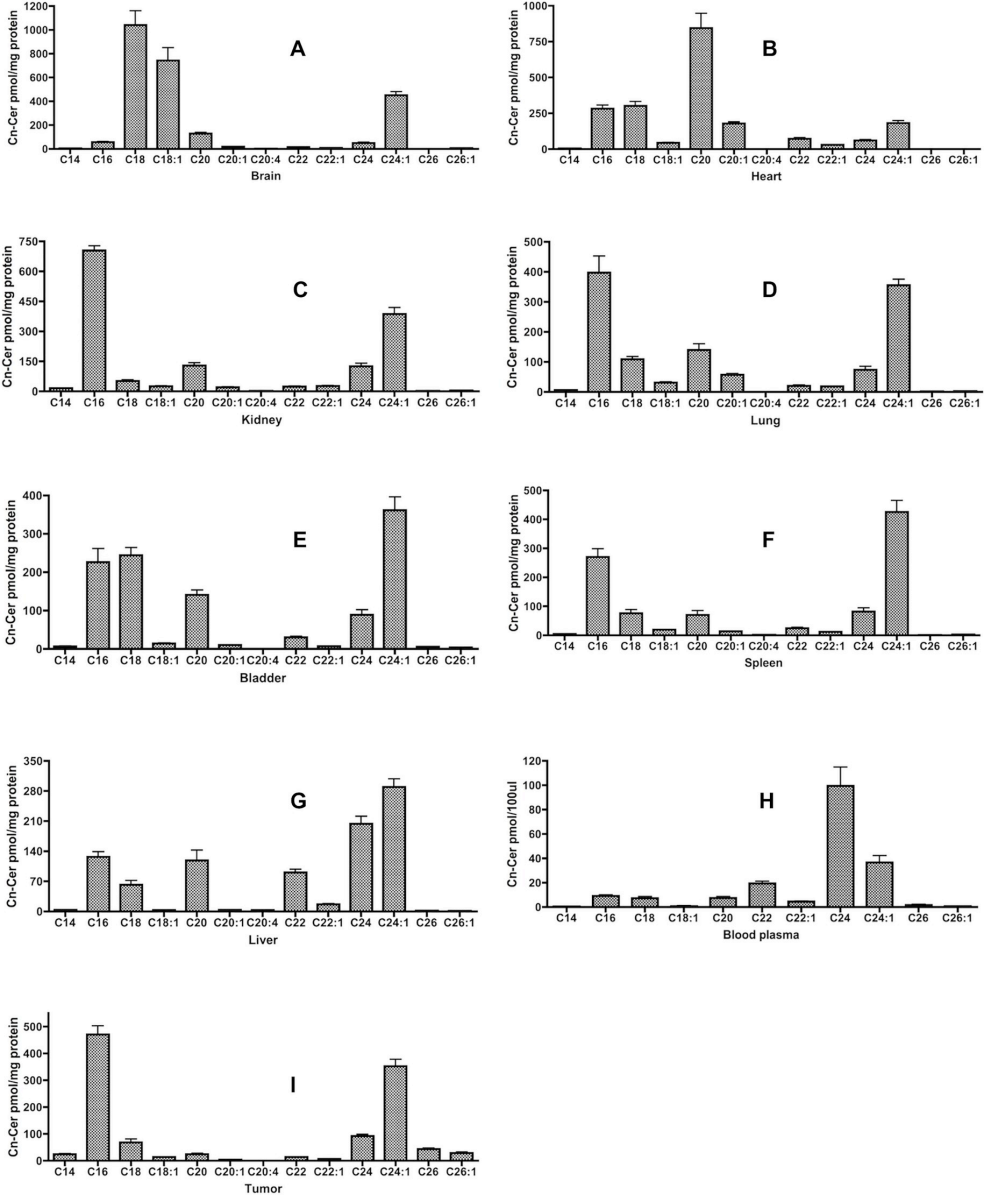
Cn-Cer’s profile in various tissues. Results are presented as pmol Cn-Cer/mg protein with means ± st dev. of 6x replicates. A. Brain; B. Heart; C. Kidney; D. Lung; E. Bladder; F. Spleen; G. Liver; H. Blood plasma; I. Tumor xenograft.

As shown in Table 1, brain contained the highest level of Cer, and the major Cn-Cer species in the brain were C18, C18:1 and C24:1-Cer (Fig 3A). In detail, C18-Cer, the most abundant Cer species, contributed 41.1% of total Cer; C18:1-Cer provided 29.4%; whereas C24:1-Cer was about 18% of total Cer. Consequently, these three species accounted for 88.5% of Cer in the brain.

#### Heart

There was a total of 2.01nmol/mg protein Cer found in heart, which was the second highest among that 8 tissues that were investigated. The highest Cn-Cer species in the heart was C20-Cer (42.1%), which is usually a minor species. This was followed by C18-Cer (15.1%) and C16-Cer (14.1%). C18:1-Cer that was relatively high expressed in the brain showed very low level in the heart (2.2%) whereas C20:1-Cer that was very poorly expressed in other tissues was highly expressed in the heart (179.0±30.0 pmol, 8.9%, Fig 3B).

#### Kidney

The third highest level of Cer was found in kidney (1.52nmol/mg protein). The highest level of Cn-Cer species in the kidney was C16-Cer (46.3%), followed by C24:1-Cer (25.4%). The other species were all below 10% of total (Fig 3C).

#### Lung

There was 1.21nmol/mg protein Cn-Cer found in lung tissue. Similar to kidney, the first two highest Cn-Cer species in the lung were C16-Cer (32.7%) and C24:1-Cer (29.3%). In lung, we also detected a relatively high level of C20-Cer (11.5%, Fig 3D).

#### Bladder

There was a total of 1.14nmol/mg protein Cer found in bladder, and the most abundant Cn-Cer species was C24:1-Cer (31.7%), followed by C18-Cer (21.4%), C16-Cer (19.9%) and C20-Cer (12.4%, Fig 3E). These four major species accounted for 85.4% of Cer in the bladder.

#### Spleen

We could detect only 1.01nmol/mg protein Cer in spleen, and the most abundant Cn-Cer species was C24:1-Cer (42.3%), followed by C16-Cer (26.8%, Fig 3F). The remaining species were all below 10% of total.

#### Liver

The lowest level of Cer was found in liver, at only 0.93nmol/mg protein. Among the different Cn-Cer, the most abundant species was C24:1-Cer (31.3%), followed by C24-Cer (22%), C16-Cer (13.7%) and C20-Cer (12.8%). Interestingly, we also observed a relatively high level of C22-Cer in the liver (91.0±16.0 pmol, 9.8%) while most tissues had levels of only around 25pmol/mg protein (Fig 3G).

#### Plasma

There was a total of 188pmol Cn-Cer found in 100ul blood plasma. The most abundant Cn-Cer species in plasma was C24-Cer (53%), which was never above 10% in the other tissues that were investigated (except liver). C24:1-Cer was the second highest Cn-Cer species in plasma and contributed 19.5% of total Cer. The third major species in plasma was C22-Cer (10.5%, Fig 3H), which was only 1–3% in most of tissues. These three major species accounted for 83% of total Cer in plasma.

#### Tumor xenograft

The total Cer in tumor was 1.14nmol/mg protein, and same as kidney and lung, the top two most abundant Cn-Cer species were C16-Cer (41.2%) and C24:1-Cer (30.8%). Tumor also contained significant amounts of C26-Cer (43.5±10.1 pmol, 3.8%) and C26:1-Cer (28.8±8.9 pmol, 2.5%), which were hardly detectable in other tissues (Fig 3I).

## Discussion

To explore SL metabolism pathways that also have therapeutic benefits for cancer, we generated initial survey of bioactive SL species across the xenograft mouse tissues. Our data disclose an intricate tissue distribution of various species such that each tissue shows a unique SL profile, and most likely, the differences in levels are due to the expression levels of various SL metabolic enzymes, especially the Cer synthases [17,18]. The common Cer species that were represented in most of the tissues are C24:1-Cer (8/9) and C16-Cer (7/9). The only tissue that had C24:1-Cer below 10% is heart (9.1% of total, 182.9± 38.2 pmol). Interestingly, brain and plasma contained quite low levels of C16-Cer (2.2% in brain, 56.5±17.4 pmol; 4.9% in plasma, 11.5±2.7 pmol) as compared to the other 7 tissues that were investigated, while the highest level of C16-Cer was in kidney. We also observed 4 tissues contained relatively high level of C20-Cer whereas 3 tissues had high level of C18-Cer, and the highest level of C20-Cer was in heart, whereas C18-Cer was the most abundant Cer species in the brain.

Among all Cer species, we also detected some Cer only heavily presented in certain tissues; for instance, C18:1-Cer, which contributed only 1–2% in most of the tissues, was highly present in the brain (743.3±262.2 pmol, 29.4%); C22-Cer was one of the major Cer species in the plasma; C24-Cer was only highly present in the liver and plasma (Table 3). Furthermore, there was a total of 188pmol of Cn-Cer found in 100ul plasma, but the most abundant SL in the plasma was Sph1-P (288.4±70.5 pmol), which had only very low amounts (around 1pmol) found in the eight other tissues; Moreover, blood also contained the highest level of dhSph1-P (79.5±30.0 pmol), which had either trace mount (<0.3pmol) or below the detection limit (BDL) in the eight tissues that were investigated (Table 4).

**Table 3:** Major Cn-Cer species (>10% of total Cer) in various tissues.

Tissues (/mg pro)	**C16**	**C18**	**C18:1**	**C20**	**C22**	**C24**	**C24:1**	**% of total**
Brain		1040.9±295.5	743.3±262.2				451.5±72.9	**88.5**
Heart	282.2±63.3	301.9±76.5		844.7±252.3				**71.2**
Kidney	704.0±60.1						386.5±80.9	**71.6**
Lung	397.1±136.0			139.7±50.6			355.4±48.9	**73.5**
Tumor	471.0±78.5						352.1±64.6	**72.0**
Bladder	226.2±87.0	244.3±48.4		141.1±31.3			361.4±86.4	**85.4**
Spleen	270.1±70.3						425.7±96.6	**69.1**
Liver	127.0±29.9			118.5±58.8		203.6±43.7	289.8±45.3	**79.8**
Plasma (100ul)					24.4±5.7	124.3±66.2	45.8±17.0	**82.9**

Results are presented as pmol Cn-Cer/mg protein with means ± st dev. of 6x replicates.

**Table 4:** Levels of Sph 1-P and dhSph 1-P in various tissues.

Tissue (/mg protein) *	**Sph 1-P**	**dhSph 1-P**
Brain	0.7±1.2	0.2±0.2
Heart	0.5±0.7	BDL
Kidney	BDL	BDL
Lung	0.3±0.3	0.1±0.1
Tumor	1.1±1.5	BDL
Bladder	0.6±1.0	BDL
Spleen	1.1±1.4	0.3±0.2
Liver	0.3±0.3	BDL
Plasma (100ul)	**288.4±70.5**	**79.5±30.0**

Results are presented as pmol SL/mg protein with means ± st dev. of 6x replicates.

*BDL = below the detection limit

**Fig 4:**
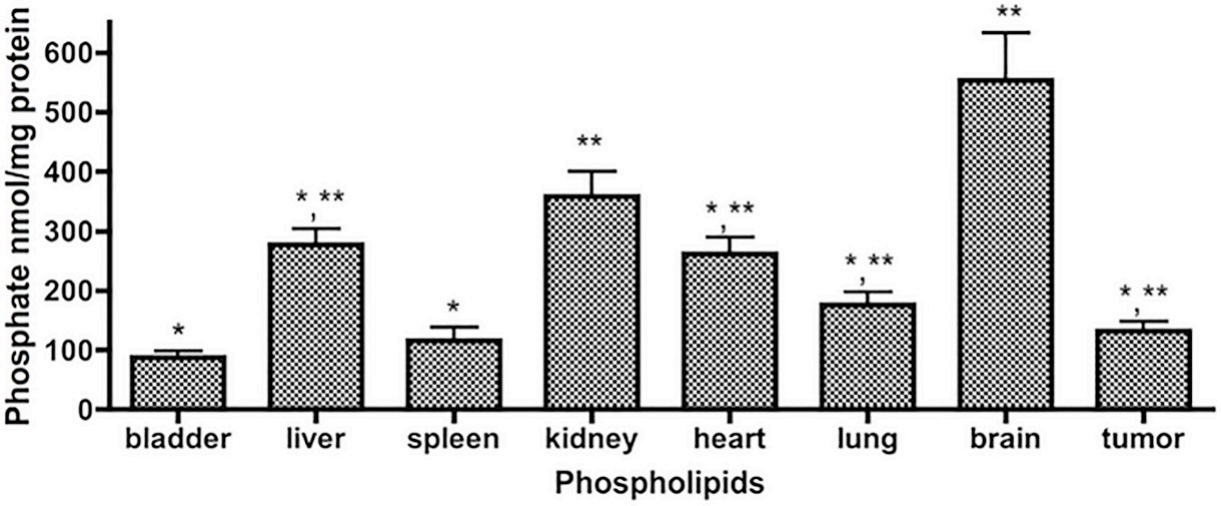
Levels of total phosphate in various tissues. Results are presented as nmol/mg protein with means ± st dev. of 6x replicates. * *p*<0.05 (*vs* brain), ** *p*<0.05 (*vs* bladder).

There have been a few studies evaluating a larger set of lipids in mouse models, but they either included few tissues or presented only major lipids. They also utilized various mouse backgrounds, depending on the goal of the study [19–22]. Comparing these studies, there are some typical differences. The first relates to data normalization, some including ours used tissue protein, others used total lipid phosphate, or directly used per wet weight of tissues. Based on that, we also measured the level of total phosphate of each sample that had been quantified with same amount of protein (800ug), and the results indicated their phosphate were not equal, the difference between the highest (brain) and the lowest (bladder) was over 6 fold (Fig 4). The conversion between per mg protein to per nmol phosphate is obtained via dividing the results by the following: bladder (87.0); liver (276.7); spleen (115.7); kidney (357.8); heart (262.1); lung (176.3); brain (552.5), and xenograft tumor (131.8). The second issue relates to methods of extracting lipids. Some used Bligh and Dyer (B&D) extraction [23], while we used methods that were developed by Bielawski et al, and the difference between B&D extraction and our extraction have been published [24]. Therefore, due to these differences, it is hard to compare various sets of results directly. However, beyond these, we did note some interesting points; for example, there are tremendous amounts of C18:1-Cer in nude mice brain (C18:C18:1-Cer, 1.4:1.0), while there was actually very low level of C18:1-Cer present in C57BL/6J x FVB brain (C18:C18:1-Cer, 179.0:1.0).

While this resource represented an initial SL exploration in xenograft mouse model, it has limitations, such as the extraction and analytic methods were selected to study the profile of Cer/Sph/Sph 1-P, therefore, we did not evaluate complex SL at this time.
